# Metabolic phenotype of bovine blood-derived neutrophils is altered in milk

**DOI:** 10.1038/s41598-025-93929-y

**Published:** 2025-03-19

**Authors:** Heidi C. Duda, Carolin J. Sprenzel, Andrea Didier, Armin M. Scholz, Cornelia A. Deeg, Roxane L. Degroote

**Affiliations:** 1 Chair of Physiology, Department of Veterinary Sciences, Ludwig-Maximilians-University Munich, 82152 Martinsried, Germany; 2 Chair of Hygiene and Technology of Milk, Department of Veterinary Sciences, Ludwig-Maximilians-University Munich, 85764 Oberschleißheim, Germany; 3 Livestock Center of the Faculty of Veterinary Medicine, Ludwig-Maximilians-University Munich, 85764 Oberschleißheim, Germany

**Keywords:** Inflammation, Neutrophils, Energy metabolism, MHC class II, Apoptosis

## Abstract

In a healthy udder, immune cells from the peripheral bloodstream migrate into mammary tissue in low numbers to provide baseline immune surveillance, without triggering inflammation. In bovine intramammary inflammation, on the other hand, high amounts of leukocytes are recruited, causing severe inflammation. We were interested in leukocyte subpopulations and functional differences between blood- and milk-derived neutrophils from healthy and inflamed udder quarters. In this context, we found a distinct leukocyte subpopulation profile dependent on the health status of mammary gland quarters, with a predominant T cells population in heathy mammary gland quarters and a shift to macrophages and granulocytes in inflammation. Further, we detected divergent expression of major histocompatibility complex class II and interleukin 2 receptor CD25 on the surface of milk- and blood-derived neutrophils, pointing to antigen presentation and immune modulatory properties. Moreover, we observed differences in production of reactive oxygen species, deviant early and late apoptosis and functional changes in these cells, pointing to an altered metabolic phenotype in milk cells dependent on the health status of mammary gland quarters. These findings provide insights into the functional adaptations of neutrophils in different environments, highlighting the importance of metabolic alterations for immune cell function.

## Introduction

During intramammary inflammation, elevated levels of pro-inflammatory cytokines and chemokines increase permeability of the blood-milk barrier and recruit leukocytes, mainly neutrophils, from the peripheral bloodstream^1–3^. Neutrophils are highly effective cells of the innate immune system and are essential for clearance of pathogens from the inflamed mammary gland through various effector functions, including phagocytosis, degranulation, production of reactive oxygen species (ROS) and neutrophil extracellular trap release^4,5^. To limit tissue damage caused by excessive effector functions, neutrophils undergo apoptosis after several functional cycles and are subsequently cleared by macrophages^4,6^.

Neutrophil migration and effector functions, and thus their ability to efficiently eliminate pathogens, are influenced by mitochondrial metabolism, despite neutrophils primarily generating adenosine triphosphate (ATP) through glycolysis^7,8^. Inhibition of neutrophil mitochondria has been shown to reduce ROS production, neutrophil extracellular trap release, migration speed and chemotaxis^7,9,10^. Consequently, mitochondrial metabolism of neutrophils represents a critical factor in both inflammation and pathogen elimination^7^.

Although it is evident that neutrophils infiltrate the inflamed mammary gland for elimination of pathogens, thereby sustaining or even promoting inflammation, these cells may also participate in the regulation of inflammatory mechanisms, taking on an immunomodulatory role^11,12^. Additionally to the standard neutrophil effector function repertoire, regulatory neutrophils are capable of suppressing T cell proliferation through mechanisms involving ROS production and direct cell-cell interactions^11,13^. For instance, superoxide generated by the NADPH oxidase complex in neutrophils is converted into hydrogen peroxide by superoxide dismutase, which suppresses T cell proliferation by, among other mechanisms, inducing apoptosis and inhibiting Nf-κB activation, a key pathway for T cell activation^13,14^.

Furthermore, the formation of an immunological synapse between T cells and regulatory neutrophils can also contribute to suppression of T cell proliferation^11^. Various surface molecules of neutrophils such as CD11b, major histocompatibility complex (MHC) class II and programmed death ligand 1 play a supporting role in this process^11,15,16^. Interestingly, neutrophils also have the ability to present antigens to T cells, which can have the opposite effect and stimulate T cell activation^17–19^. In bovines, neutrophils expressing MHC class II have recently been shown to suppress T cell proliferation^11,20^. However, the presence and potential role of bovine regulatory neutrophils in inflammation, particularly in intramammary inflammations, remains largely unexplored.

Therefore, our study aimed at characterizing milk cells, mainly neutrophils, from healthy and inflamed mammary gland quarters in comparison to blood-derived neutrophils, with regard to their immune effector functions and metabolic phenotype.

## Results

### Blood cells and milk cells from healthy and inflamed udder quarters comprise divergent leukocyte subpopulations

**Fig. 1:**
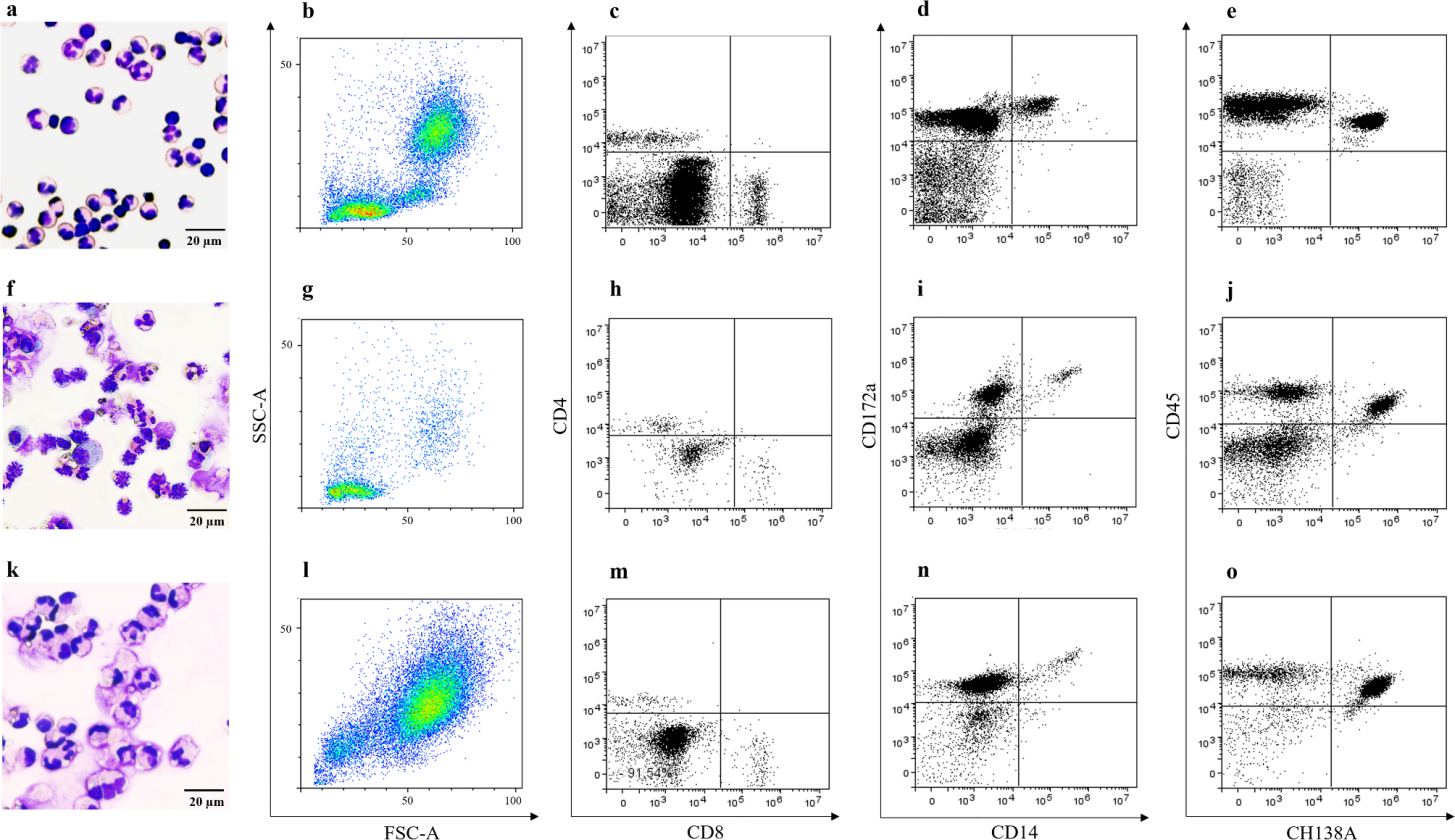
Characterization of immune cells in blood (**a**–**e**) and milk samples from healthy (**f**–**j**) and inflamed udder quarters (**k**–**o**). Representative cytospin preparations of blood cells (**a**) and milk cells from healthy (**f**) and inflamed (**k**) udder quarters in Haema Quick staining visualises different cell populations and morphology. Representative SSC/FSC density plots of viable CD45^+^ blood leukocytes (**b**) and viable CD45^+^ milk leukocytes from healthy (g) and inflamed (l) udder quarters demonstrate divergent leukocyte subpopulations. Representative dot plots of CD4/CD8 (**c**,**h**,**m**), CD172a/CD14 (**d**,**i**,**n**), and CD45/CH138A (**e**,**j**,**o**) staining show differences within the leukocyte subpopulations between blood cells (**c**–**e**) and milk cells from healthy (**h**–**j**) and inflamed (**m**–**o**) udder quarters in more detail.

Using flow cytometry analysis, we detected a divergent composition of viable CD45^+^ leukocyte subpopulations between blood cells and milk cells from both healthy and infected udder quarters (Fig. 1). Blood-derived cells showed apparent lymphocyte, monocyte and granulocyte populations (Fig. 1a,b). In healthy mammary gland quarters, lymphocytes predominated, while only a few granulocytes and macrophages were visible (Fig. 1f,g). In inflamed quarters, on the other hand, the main leukocyte population was comprised of granulocytes (Fig. 1k,l).

Investigating these differences in more detail, we could show that the percentage of CD4^+^ T lymphocytes and CD8^+^ T lymphocytes was significantly higher in healthy udder quarters than in blood and in inflamed udder quarters (Table 1; Fig. 1c,h,m). Furthermore, we detected a significantly lower C4^+^:CD8^+^ ratio in inflamed udder quarters compared to blood and healthy udder quarters (Supplementary Fig. 2). Additionally, the percentage of monocytes/macrophages (CD172a^+^CD14^+^) was significantly lower in inflamed udder quarters compared to blood (Table 1; Fig. 1d,i,n). Finally, we observed a higher percentage of neutrophils (CH138A^+^) in inflamed udder quarters compared to blood and healthy udder quarters (Table 1; Fig. 1e,j,o).

**Table 1:** Percentage (mean value ± standard deviation) and statistical differences (*p*-value) of leukocyte subpopulations in blood and milk from healthy and inflamed udder quarters.

Surface antigen	Comparison group 1	Comparison group 2	*p*-value	
	Blood	Healthy udder quarter		
CD45^+^CD4^+^CD8^-^	5.39 ± 1.33	23.13 ± 10.81	0.016	*
CD45^+^CD4^-^CD8^+^	4.32 ± 0.94	18.50 ± 11.93	0.016	*
CD45^+^CD172a^+^CD14^+^	6.01 ± 1.51	4.12 ± 2.47	0.093	
CD45^+^CH138A^+^	47.30 ± 10.12	35.21 ± 20.11	0.157	
	Blood	Inflamed udder quarter		
CD45^+^CD4^+^CD8^-^	5.39 ± 1.33	3.32 ± 3.46	0.247	
CD45^+^CD4^-^CD8^+^	4.32 ± 0.94	4.52 ± 3.87	0.917	
CD45^+^CD172a^+^CD14^+^	6.01 ± 1.51	2.37 ± 1.89	0.0008	***
CD45^+^CH138A^+^	47.30 ± 10.12	67.06 ± 17.66	0.016	*
	Healthy udder quarter	Inflamed udder quarter		
CD45^+^CD4^+^CD8^-^	23.13 ± 10.81	3.32 ± 3.46	0.016	*
CD45^+^CD4^-^CD8^+^	18.50 ± 11.93	4.52 ± 3.87	0.032	*
CD45^+^CD172a^+^CD14^+^	4.12 ± 2.47	2.37 ± 1.89	0.144	
CD45^+^CH138A^+^	35.21 ± 20.11	67.06 ± 17.66	0.006	**

Interestingly, we observed a divergent morphology of blood cells and milk cells from healthy and inflamed udder quarters (Fig. 1a,f,k). Milk-derived leukocytes showed increased cytoplasm-to-nucleus ratio, and a blurred appearance of the plasma membrane compared to cells from blood. These properties might indicate increased deformability, adhesion and activation of these cells. Additionally, we observed cytoplasmic vacuoles in milk-derived neutrophils, especially in those from inflamed udder quarters (Fig. 1f,k).

### High expression of MHC class II and CD25 on neutrophils from healthy udder quarters points to antigen presentation and increased activation

**Fig. 2:**
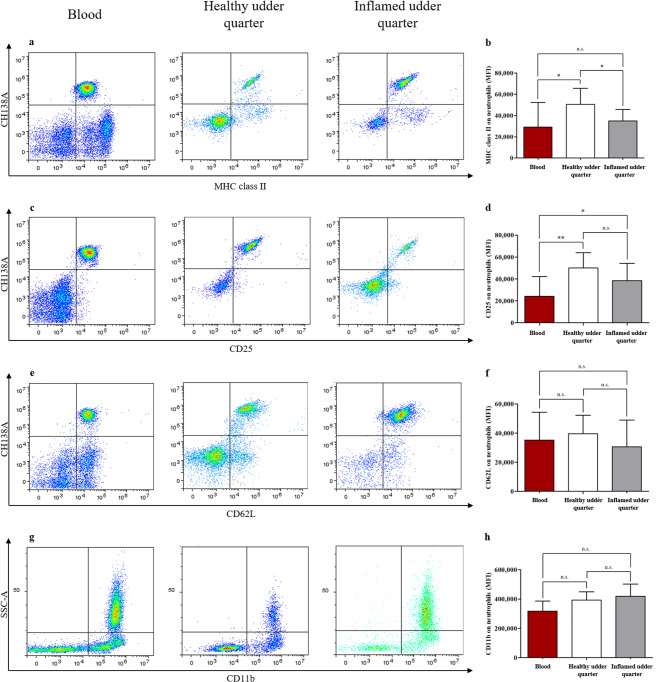
Comparison of MHC class II, CD25, CD62L and CD11b expression on neutrophils from blood and milk. Representative density plots of MHC class II (**a**) and CD25 (**c**) staining visualize differences in the expression of these molecules on the surface of blood- and milk-derived neutrophils from healthy and inflamed udder quarters. Representative density plots of CD62L (**e**) and CD11b (**g**) staining show no expression differences on the surface of blood- and milk-derived neutrophils from healthy and inflamed udder quarters. (**b**) Milk neutrophils from healthy udder quarters showed a significantly (*p ≤ 0.05) higher expression of MHC class II compared to blood neutrophils and milk neutrophils from infected udder quarters. (**d**) Milk neutrophils from healthy and infected udder quarters showed a significantly (**p ≤ 0.01, *p ≤ 0.05) higher expression of CD25 compared to blood neutrophils. No significant differences in the expression of CD62L (**f**) and CD11b (**h**) on the surface of blood- and milk-derived neutrophils were observed.

In order to investigate the neutrophil subset in more detail and gain insights on possible changes in activation, the expression of surface receptors CD11b, CD62L, MHC class II and CD25 was analyzed using flow cytometry. We observed no significant differences in the expression of CD62L (Fig. 2e,f) and CD11b (Fig. 2g,h) on the surface of blood- and milk-derived neutrophils. Contrary to this, we detected high levels of MHC class II on milk-derived neutrophils from healthy udder quarters, and a significant decrease of this molecule on both blood-derived neutrophils and neutrophils from inflamed udder quarters (Fig. 2a,b). Similarly, CD25 was highly expressed on the surface of neutrophils from healthy udder quarters with significant decrease of this molecule on blood-derived neutrophils, but no significant expression changes compared to neutrophils from inflamed quarters. However, we observed significant differences in CD25 expression between neutrophils from inflamed udder quarters and those from blood. (Fig. 2c,d). These differences in CD25 and MHC class II expression may point to activation and increased antigen presentation of milk-derived neutrophils from healthy udder quarters.

### Decreased ROS production and activation after PMA stimulation correlates with increased apoptosis rate in milk-derived cells

**Fig. 3:**
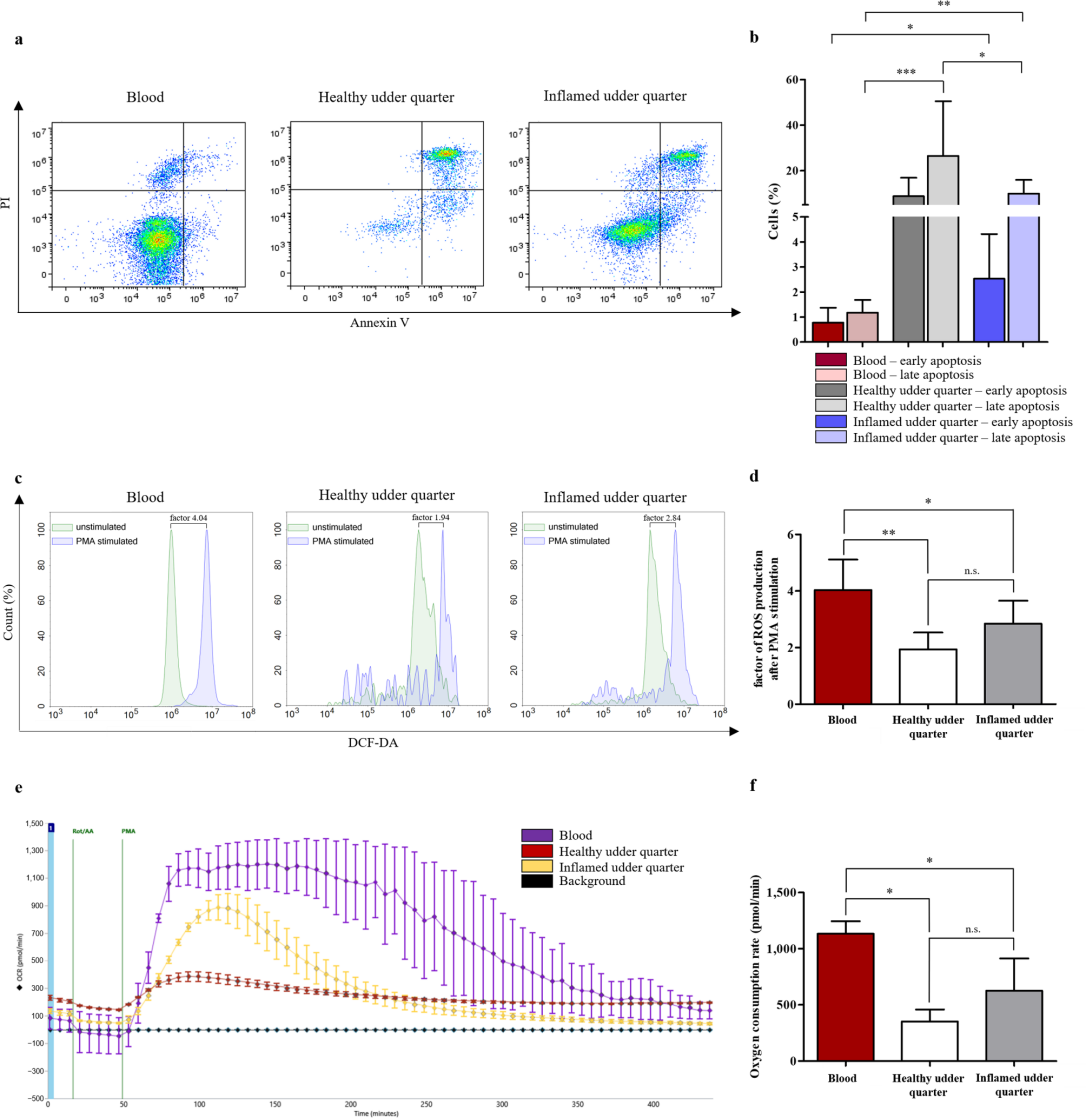
Comparison of early and late apoptosis rate of neutrophils in blood, healthy udder quarter and inflamed udder quarter and ROS production and OCR in neutrophils from blood and milk. (**a**) Representative density plots of Annexin V/PI stained blood- and milk-derived neutrophils from healthy and inflamed udder quarters. (**b**) Milk neutrophils from infected udder quarters showed a significantly (*p ≤ 0.05) higher early apoptosis rate compared to blood neutrophils. Milk neutrophils from healthy and infected udder quarters exhibited a significantly (***p ≤ 0.001, **p ≤ 0.01) higher late apoptosis rate than blood neutrophils. The late apoptosis rate of milk neutrophils from healthy udder quarters was significantly (*p ≤ 0.05) increased compared to milk neutrophils from infected udder quarters. (**c**) Representative histograms show DCF-DA staining of unstimulated and PMA stimulated blood- and milk-derived neutrophils from healthy and inflamed udder quarters. (**d**) DCF-DA staining revealed a significantly (**p ≤ 0.01, *p ≤ 0.05) lower ROS production of milk neutrophils after PMA stimulation compared to blood neutrophils. (**e**) Representative OCR after injection of Rot/AA and PMA. (**f**) OCR of milk-derived cells after mitochondrial inhibition and PMA stimulation was significantly (*p ≤ 0.05) lower compared to blood-derived cells.

To assess whether the expression changes of MHC class II and CD25 correlate with changes in neutrophil viability, we measured early and late apoptosis using Annexin V/PI staining on freshly obtained cells from blood and milk. Compared to blood-derived neutrophils, the early apoptosis rate (Annexin V^+^/PI^−^) of neutrophils from inflamed udder quarters was significantly increased (Fig. 3a, b). No significant difference was detected between cells from blood and healthy udder quarters as well as cells from healthy and inflamed udder quarters (Fig. 3a,b). The late apoptosis rate (Annexin V^+^/PI^+^) of milk-derived neutrophils from healthy and inflamed udder quarters was significantly increased compared to blood-derived neutrophils (Fig. 3a,b). Moreover, milk-derived neutrophils from healthy udder quarters exhibited a significantly higher late apoptosis rate than milk-derived neutrophils from inflamed udder quarters (Fig. 3a,b).

To examine further effector functions of neutrophils and a possible correlation with the observed apoptosis rates, cells were stimulated with phorbol 12-myristate 13-acetate (PMA) and ROS production was determined by flow cytometry using 2′,7′-Dichlor-dihydrofluorescein-diactetat (DCF-DA) staining, which is converted into fluorescent 2′,7′-Dichlorfluorescein upon oxidation. Compared to blood-derived neutrophils, we detected a significantly lower ROS production in neutrophils from both healthy and inflamed udder quarters (Fig. 3c,d). However, no significant differences were observed between inflamed and healthy udder quarter derived neutrophils (Fig. 3c,d).

For evaluation and quantification of the observed differences in oxidative burst response to PMA stimulation over an extended period of time, we subsequently analyzed the oxygen consumption rate (OCR) of blood- and milk-derived cells with a Seahorse XFe Analyzer after blocking mitochondrial respiration with rotenone & antimycin A (Rot/AA). Compared to blood, we detected a significantly decreased OCR of milk cells from both healthy and inflamed udder quarters after PMA stimulation (Fig. 3e,f). The OCR did not differ between cells from healthy and inflamed udder quarters (Fig. 3e,f).

Interestingly, the decreased ROS production and OCR in PMA stimulated, milk-derived cells correlated with increased apoptosis rate observed in freshly isolated cells, respectively.

### Changed mitochondrial function of blood- and milk-derived leukocytes points to a divergent metabolic phenotype

Since we identified significant differences in milk-derived neutrophil activation and apoptosis rates, we were now interested to see if we could also detect differences in mitochondrial function between blood and milk cells from healthy and inflamed udder quarters. To maintain the most in vivo-like functional entity regarding leukocyte subset composition, we analyzed leukocytes including all subsets present in the samples.

**Fig. 4:**
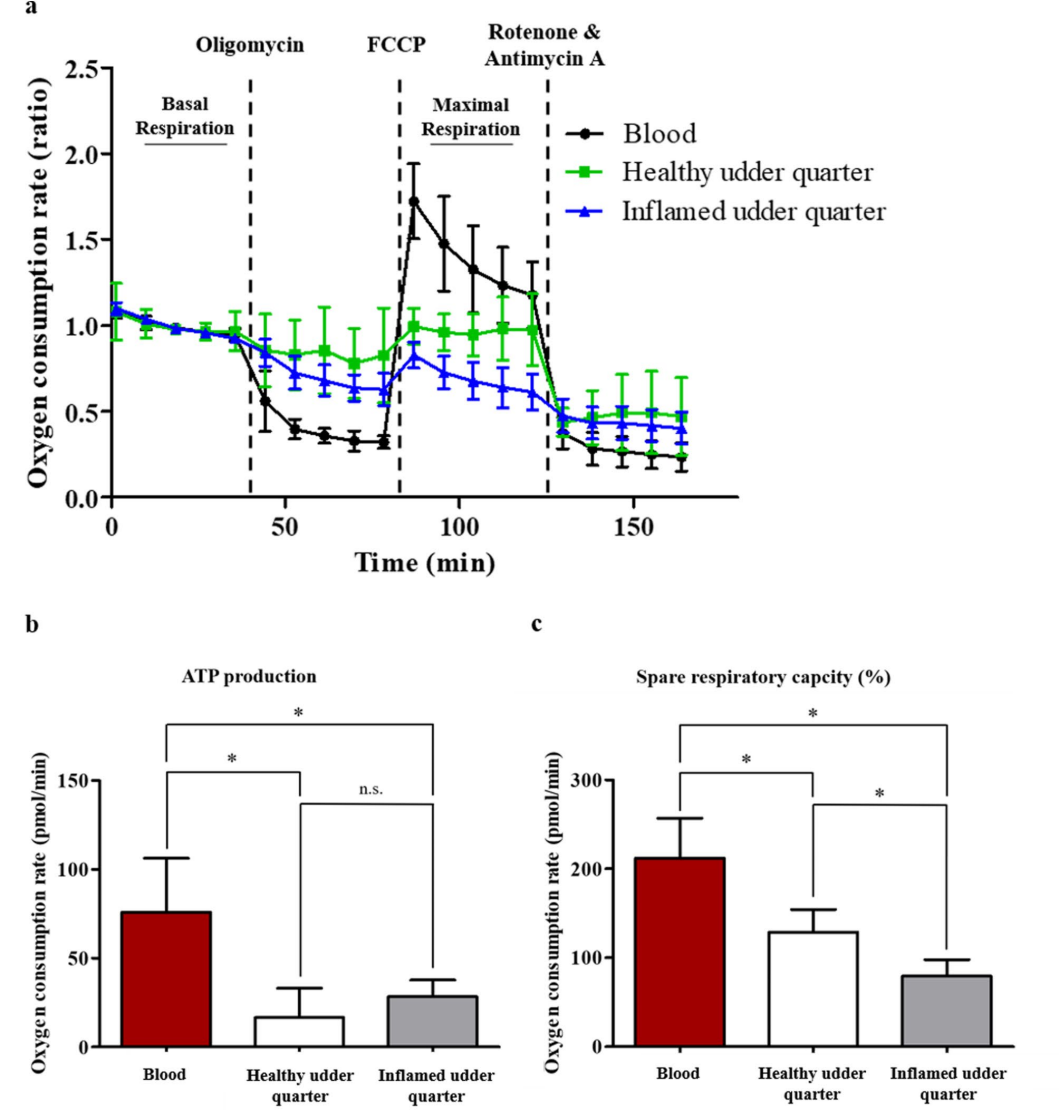
Mitochondrial function of blood- and milk-derived cells. (**a**) Mitochondrial metabolism of blood-derived cells (black) and milk-derived cells from healthy (green) and inflamed (blue) udder quarters after injection of Oligomycin, FCCP and Rot/AA. Ratios were determined after normalizing each measurement against the baseline mean. Data are presented as mean ± SD. (**b**) ATP production is significantly (*p ≤ 0.05) lower in milk-derived cells compared to blood-derived cells after Oligomycin induced inhibition of complex V of the respiratory chain. (**c**) The spare respiratory capacity of milk-derived cells is significantly (*p ≤ 0.05) decreased compared to blood-derived cells. Milk-derived cells from inflamed udder quarters showed a significantly (*p ≤ 0.05) lower spare respiratory capacity compared to healthy udder quarters.

*Via* the Seahorse XF Cell Mito Stress Assay we first measured basal respiration (Fig. 4a, far left section). After the inhibition of the ATP synthase (complex V) of the respiratory chain reaction through injection of oligomycin, we observed a decrease in the amount of oxygen used through ATP production (Fig. 4a, left section). In detail, milk-derived leukocytes from healthy and inflamed udder quarters both showed a significantly decreased ATP-linked respiration compared to blood cells (Fig. 4b). No differences were observed between healthy and inflamed udder quarters (Fig. 4b).

Measurement of the maximal respiration after the injection of the uncoupler carbonyl cyanide-4 (trifluoromethoxy) phenylhydrazone (FCCP) (Fig. 4a, right section), and calculation of the difference between basal and maximal respiration, revealed a significant decrease in the spare respiratory capacity of leukocytes from healthy and inflamed udder quarters compared to blood-derived cells (Fig. 4c). Additionally, the spare respiratory capacity significantly decreased in leukocytes from inflamed udder quarters compared to healthy udder quarters (Fig. 4c). The spare respiratory capacity indicates the ability of the cells to respond to changes in energetic demand.

Finally, blocking of mitochondrial respiration through a combination of rotenone, a complex I inhibitor, and antimycin A, a complex III inhibitor, had no significant effect on nonmitochondrial oxygen consumption (Fig. 4a, far right section).

## Discussion

The mammary gland is habitually infiltrated by small numbers of immune cells from the bloodstream, ensuring baseline immune surveillance^21^. However, during bovine intramammary inflammation, a substantial influx of leukocytes occurs, resulting in pronounced inflammation^21^. In our study, we detected a divergent composition of viable leukocyte subpopulations in blood, milk from healthy udder quarters and milk from inflamed udder quarters. As physiologically expected, blood-derived leukocyte subsets showed a slightly more lymphocytic phenotype, whereas healthy mammary gland quarters were clearly dominated by CD4^+^ and CD8^+^ T cells. Contrary to this, cells from inflamed quarters were mainly neutrophils, which is in line with previous studies described increased neutrophil infiltration in intramammary inflammation^22–25^. Since we were interested in gaining more insights in the impact of these different environments on the functional properties of neutrophils, we analyzed markers for activation and antigen presentation.

CD11b and CD62L, both activation markers of neutrophils^26^, did not significantly differ in abundance between blood, healthy udder quarters and inflamed udder quarters. This suggests that at the time of sampling, the activation status of the investigated neutrophils was unchanged between the different extracellular environments. To determine whether we missed peak activation, more experiments are needed comparing cells obtained from different sampling times.

The expression of MHC class II on the surface of bovine blood and milk neutrophils was previously described only on a subset of neutrophils^11,20,27^, whereas we observed an expression on almost all neutrophils in our analysis. Interestingly, MHC class II expression on neutrophils suggests a role of these cells as atypical antigen presenting cells^17–19^ and in the regulation of the adaptive immune response, for example via suppression of T cell proliferation^11^. The increased expression of MHC class II on neutrophils from healthy udder quarters may therefore be an indication for immune modulatory properties, as shown in other studies on bovine neutrophils^11,20^, underlining the role of these cells in providing immune surveillance in the healthy udder. The decrease in inflamed udder quarters suggests that inhibitory properties may be decreased due to functional adaptation in course of inflammation, for example as a reaction to causative agents^28,29^.

Similar to MHC class II, the surface expression of the Interleukin-2 (IL-2) receptor CD25 was highest on neutrophils from healthy udder quarters. However, the slight decrease observed in milk-derived neutrophils from quarters with intramammary inflammation was not significant. Although CD25 is typically associated with regulatory T cells^30^, it has previously been identified on bovine neutrophils, suggesting this molecule to be a potential biomarker for inflammations in cattle^31^. Contrary to our studies, CD25 expression was compared on mRNA level between healthy cows and cows with extra- and intramammary infections and showed strong correlation with the severity of disease^31^. Studies on human neutrophils previously described unchanged expression levels of the IL-2 receptor CD25 after stimulation with granulocyte-macrophage colony-stimulating factor, while IL-2-binding increased^32^, suggesting that effective binding of IL-2 is not correlated to the intensity of CD25 receptor expression. Other studies showed that recombinant IL-2 can induce production of ROS and thereby enhance the bacterial killing rate of neutrophils in milk^33,34^; pointing to increased activation through ligation of CD25 by IL-2. Further studies are needed to clarify whether CD25 is associated to the activation status of bovine neutrophils or possibly plays a role in immune regulation similar to regulatory T cells, or if the manifestation of these characteristics depends on the extracellular environment these cells are exposed to.

Although we observed an upregulation of CD25 on milk-derived neutrophils suggesting an activation of effector functions *via* IL-2, we found a decreased oxidative burst response of PMA-stimulated neutrophils in milk compared to blood, which has similarly been shown in previous investigations on healthy cows and goats^35,36^. This leads us to suspect that, despite increased CD25 expression, IL-2 was not able to trigger increased ROS production in the milk-derived neutrophils. This may simply originate in insufficient levels of IL-2 in the milk samples that we used for analysis or have other underlying reasons, such as the specific intra-udder environment and the impact of the diverse variety of milk-derived molecules on ROS production. For instance, a reduced oxidative burst of bovine milk neutrophils was observed after diapedesis across mammary epithelium in vitro^37^. Furthermore, milk components, such as fat and casein, have been shown to inhibit phagocytosis and oxidative burst response of bovine milk neutrophils^38^. Moreover, the decreased ROS production that we showed in milk cells may have occurred due to cell exhaustion caused by a pre-existing excessive ROS production in course of activation after migration into the udder. This is supported by the observation of cytoplasmic vacuoles in freshly isolated, unstimulated milk-derived neutrophils in our study, which are indicative of ongoing ROS production in these cells^39^. The fact that these cells may initially already be in an advanced state of activation, might explain the higher rates of late apoptosis that we observed directly after isolation. These lower viability rates of neutrophils from milk compared to neutrophils from the peripheral bloodstream are not only in line with other studies on milk-derived neutrophils of cows and goats^35,40–42^, but also underline the fact that diapedesis through the blood-milk barrier is an initial trigger for neutrophil apoptosis after activation^43^.

Moreover, we identified a correlation of apoptosis rate and udder quarter health. To date, there is no consensus on the correlation of neutrophil viability with udder quarter health status. Several studies on the viability and apoptosis rate of neutrophils in milk clearly support our findings of increased apoptosis in healthy udder quarters^41,44^, whereas others rather describe a higher rate of viable neutrophils, therefore less apoptotic cells in healthy udder quarters compared to diseased udder quarters^24,45,46^. The studies indicating reduced apoptosis in healthy udder quarters, however, lack uniform cell preparation methods and viability or apoptosis rate analysis of neutrophils, which needs to be kept in mind when interpreting respective results. In addition, heterogeneous definitions were used regarding the mammary gland health^24,41,45^.

Controlled apoptosis of neutrophils and their subsequent phagocytosis by macrophages is an important process in reducing tissue damage of the mammary gland and curing intramammary inflammations^6^. Inflammatory mediators regulate cell apoptosis through pro-apoptotic and anti-apoptotic signals^6^. The pro-inflammatory cytokine Tumor necrosis factor (TNF)-α is upregulated during intramammary inflammation and induces bovine neutrophils to undergo apoptosis^47,48^. In contrast, LPS stimulates toll-like receptor 4, which suppresses apoptosis of neutrophils in cows^49^. During mastitis, further cytokines are upregulated in milk, including IL-6 and IL-8, that delay programmed cell death^6,50,51^. They are responsible for mediating acute phase response and the accumulation of leukocytes in inflamed mammary glands^50,51^. In addition to these effects, an increased number of apoptosis-inhibiting mediators in inflamed udder quarters might lead to the observed lower rate of apoptotic neutrophils in our study. Further, a previous study showed that pathogens triggering intramammary inflammation can inhibit neutrophil apoptosis^5^. This may be an additional reason for the higher rate of apoptotic neutrophils in the healthy, uninflamed udder quarters. Although the healthy and the inflamed quarters are located in direct proximity, neutrophils from the healthy quarters showed a significantly higher rate of late apoptosis. We therefore assumed that the regulation of apoptosis in healthy udder quarters is not affected by the neighboring inflammation, which indicates immunological independence of the mammary gland quarters. Delayed apoptosis is important for combating the pathogen, but it also poses a risk of tissue damage. Proper regulation of apoptosis is therefore important for curing intramammary inflammation, making targeted regulation of apoptosis a potential therapeutic approach. Therefore, the mechanisms accelerating or suppressing neutrophil apoptosis need further investigations.

To our knowledge, we are the first to analyze the metabolic phenotype of bovine milk-derived leukocytes. We could show that, despite high CD25 expression suggesting increased activation, milk cells from both healthy and inflamed mammary gland quarters showed a decreased ATP production by oxidative phosphorylation compared to blood cells, which indicates energetic stress of these cells leading to restricted cellular respiration and energy deficits. Mitochondrial dysfunctions and damage during inflammations can be caused by various factors, including oxidative stress and cytokines^52,53^. In vitro studies with endometrial epithelial cells observed mitochondrial dysfunction with decreased ATP production induced by *Escherichia coli* in cattle as well as mitochondrial damage caused by *Staphylococcus aureus* in goats, both common causative agents of intramammary inflammation^53,54^. The presence of pathogens could therefore be an explanation for decreased mitochondrial ATP production of milk cells from inflamed quarters. However, since we also observed this effect in the healthy, uninflamed quarters, the presence of bacteria alone cannot be the only reason. In humans, the pro-inflammatory cytokine TNF-α induces mitochondrial dysfunction with reduced ATP production^55^. TNF-α is also released in high amounts during bovine mastitis and induces an increase in neutrophil ROS production, as already shown in humans^56,57^. We therefore hypothesize that pro-inflammatory cytokines and the oxidative stress resulting from elevated ROS production reduce mitochondrial ATP production and that this affects both the healthy and the inflamed mammary gland quarter, which suggests an immunological dependence of the quarters. In contrast, our results showed an independence of the quarters by the regulation of apoptosis. Whether and to what extent there is an immunological dependence or independence of mammary gland quarters requires further research.

Further investigations of mitochondrial functions revealed a lower spare respiratory capacity – determined by the difference of maximal and basal respiration - in milk cells compared to blood cells, indicating a reduced adaptability to increased energy demand. We suspected that this result is caused by the exhaustion of the cells due to increased energy consumption during diapedesis, as described for human neutrophils^58,59^. Additionally, we observed a correlation of spare respiratory capacity and inflammation status of mammary gland quarters. Cells from inflamed udder quarters showed a lower spare respiratory capacity compared to cells from healthy quarters. In inflamed quarters, cells consisted mainly of neutrophils, which are energy-dependent in their effector functions^60^. These energy-demanding processes and the aspects causing mitochondrial dysfunction and damage during inflammation, as previously mentioned, might lead to cell exhaustion, reflected in reduced spare respiratory capacity. While interpreting these results, however, we need to keep in mind that the milk and blood samples that were used for mitochondrial metabolism analysis comprised divergent leukocyte subsets, which may also have an impact on the analysis of metabolic functions. Moreover, the cell-cell interactions may influence cell metabolism and activation, as previously shown for γδ T cells, an abundant lymphocyte subset in cattle which has been described to inhibit CD4^+^ and CD8^+^ T cell proliferation via IL-10 secretion^61^, and was shown to increase in course of experimentally induced inflammatory response to *Streptococcus uberis* in the udder^62^. To address these limitations, more experiments are needed analyzing mitochondrial function and metabolic phenotype of isolated leukocyte subsets in milk and blood. Especially the metabolic features of pure neutrophils may be useful to gain further insights into the interplay of inflammatory environment and metabolic adaptation of these cells. Current strategies for antibody-free isolation of pure neutrophil subsets from bovine blood are promising^63^. Their suitability for the use of the obtained cells in subsequent metabolic analyses, however, remains to be determined, since neutrophils are prone to activation and the isolation process for obtaining pure neutrophils without triggering activation and subsequent functional alterations impacting the outcome of metabolic analyses is challenging. Nevertheless, our findings on an altered metabolic phenotype of milk-derived cells in inflamed mammary gland quarters provide an opportunity for further research, also with regard to therapeutic approaches.

Taken together, our findings point to a significant alteration in immunological function and metabolic phenotype of milk-derived neutrophils compared to neutrophils from the peripheral bloodstream. In addition to a possible immunomodulatory role, we described a reduced respiratory capacity of milk leukocytes, specifically from inflamed udder quarters, indicating exhaustion of these cells leading to a reduced performance of their immunological functions. Overall, our study provides a basis for further investigations concerning the impact of the metabolic phenotype on the immune response of neutrophils from milk.

## Methods

### Animals and sample collection

In this study, samples from 15 dairy cows were used (breed: Brown Swiss (*n* = 12) and Simmental (*n* = 3); lactation stages: early lactation (*n* = 5), mid lactation (*n* = 1) and late lactation (*n* = 9)). All cows showed intramammary inflammation in at least one udder quarter, determined with positive California mastitis test (CMT, SCC threshold 400,000 cells/ml) and bacteriological analysis. From each cow, milk samples from healthy and inflamed udder quarters were collected in sterile tubes. Teats were cleaned and disinfected with 70% ethanol and the first milk jets were discarded prior to sampling. Udder quarters with positive results in the CMT and in the bacteriological analysis were categorized as inflamed (*n* = 14), whereas udder quarters with negative results in both tests were grouped as healthy (*n* = 13). Heparinized (50 I.U./ml blood, Ratiopharm, Ulm, Germany) venous whole blood was drawn from the jugular vein. Sampling of blood by venipuncture and experimental approach were approved by the local authority, the Government of Upper Bavaria, permit no. ROB-55.2-2532.Vet_03-22-38. No experimental animals were used in this study. All animals were kept for the purpose of milk production. Permission from the dairy farms to use the blood samples from their animals for study purposes was obtained. All methods were carried out in accordance with relevant guidelines and regulations and are reported in accordance with ARRIVE (Animal Research: Reporting In Vivo Experiments) guidelines.

### Sample preparation

After centrifugation (room temperature, 500×*g*, 20 min, brake off) of whole blood samples, the plasma was discarded. In the remaining blood cells at the bottom of the tube, erythrocytes were removed by 30 s sodium chloride (0.2% NaCl) lysis. Isotonicity of samples was restored through addition of equal parts 1.6% NaCl. After washing (room temperature, 400×*g*, 10 min) the remaining leukocytes were briefly stored in phosphate buffered saline (PBS; NaCl 136.9 mM (Sigma-Aldrich, Taufkirchen, Germany), Na_2_HPO_4_ × 2H_2_O 8.1 mM (AppliChem, Darmstadt, Germany), KH_2_PO_4_ 1.4 mM (Sigma-Aldrich), KCl 2.6 mM (Sigma-Aldrich); pH 7.4) for further analysis. Milk samples were centrifuged at 500×*g* for 20 min at room temperature. After removal of the fat layer and the supernatant, the remaining milk cells were washed twice with PBS before analysis. All cells were counted with trypan blue solution (VWR Life Science, Darmstadt, Germany). Blood- and milk-derived leukocytes were either used the same day within two hours after isolation (cytospin preparation: cell morphology; flow cytometry analysis: apoptosis rates and ROS production after PMA stimulation; Seahorse XFe Analyzer: activation after PMA stimulation) or stored in RPMI 1640 (PanBiotech, Aidenbach, Germany) with 1% penicillin-streptomycin (PanBiotech) and 10% heat-inactivated fetal bovine serum (Sigma-Aldrich, Darmstadt, Germany) at 4 °C overnight, until further processing after approximately 20 h (flow cytometry analysis: surface markers; Seahorse XFe Analyzer: mitochondrial metabolism assay).

### Cytospin preparation

1×10^5^ cells from blood or milk with divergent udder health status were applied to a slide and centrifuged by 300×*g* for 10 min using a cytocentrifuge (Eppendorf, Wesseling-Berzdorf, Germany). Cytospin preparations were stained with Diff-Quick (Heama Schnellfärbung Diff Quick, Eberhard Lehmann GmbH, Berlin, Germany) according to the manufacturer’s specifications. The visualization was performed with a Leica DMi8 microscope, and LASX software, version 3.4.2 (both Leica, Wetzlar, Germany).

### Flow cytometry analyses

Blood cells (*n* = 8) and milk cells from healthy (*n* = 7) and inflamed (*n* = 8) udder quarters were stained in 96-well round-bottom plates with 5 × 10^5^ cells per well. All antibodies were incubated at 4°C for 20 min. For the characterization of surface antigens of leukocytes, all cells were incubated with FITC-conjugated mouse anti-bovine CD45 (isotype IgG1, clone CC1, Bio-Rad, Feldkirchen, Germany; 1:50). The following primary antibody combinations were used: mouse anti-bovine CD4 (isotype IgG2a, clone ILA11A, Kingfisher, Saint Paul, MN, USA; 1:400) labeled with FlexAble CoraLite ^®^ Plus 555 antibody labeling kit for mouse IgG2a (ChromoTek & Proteintech Germany, Planegg-Martinsried, Germany) and mouse anti-bovine CD8 (isotype IgG1, clone CC63, Bio-Rad; 1:200), mouse anti-bovine CD172a (isotype IgG1, clone DH59B, Bio-Rad; 1:10) and biotin-conjugated mouse anti-human CD14, cross-reactive to bovine (isotype IgG2a, clone TÜK4, Bio-Rad; 1:100), mouse anti-bovine granulocytes (isotype IgM, clone CH138A, Kingfisher; 1:200) and mouse anti-equine MHC class II, cross-reactive to bovine (isotype IgG2a, in house antibody; 1:400), mouse anti-bovine CD25 (isotype IgG1, clone IL-A111, Bio-Rad; 1:200) or mouse anti-human CD62L, cross-reactive to bovine (isotype IgG2b, clone FMC46, Bio-Rad, 1:50). The primary antibody mouse anti-bovine CD11b (isotype IgG2b, clone CC126, Bio-Rad, 1:200) was not applied in any combination, whereby blood cells from only five cows and milk cells from only four healthy and seven inflamed udder quarters were used. For the staining combination CD4 and CD8, blood cells from only five cows and milk cells from only four healthy and five inflamed udder quarters were used. To CD8, CD172a and CD11b stained cells, goat anti-mouse IgG, F(ab’)_2_ fragment specific secondary antibody conjugated with Alexa Fluor 647 (Dianova, Hamburg, Germany; 1:400) was added. The CD25, MHC class II and CD62L staining was visualized using goat anti-mouse IgG (H + L) secondary antibody, Alexa 568 conjugate (Thermo Fisher Scientific, Ulm, Germany; 1:500). For the CH138A primary antibody, secondary antibody donkey anti-mouse IgM conjugated with Alexa Fluor 647 (Thermo Fisher Scientific; 1:500) was added, and for the biotin-conjugated CD14 primary antibody, Streptavidin-iFluor 555 conjugate (AAT Bioquest, Pleasanton, CA, USA; 1:300) was used. All secondary antibodies were preabsorbed with 5% heat-denatured bovine serum. Dead cells were excluded by staining with Viobility 400/452 Fixable Dye (Miltenyi Biotec, Bergisch Gladbach, Germany). The gating was performed based on the exemplary gating strategy shown in Supplementary Fig. 1.

The apoptosis rate of blood neutrophils (*n* = 8) and milk-derived neutrophils from healthy (*n* = 6) and inflamed (*n* = 8) udder quarters was determined by Annexin V-FITC/PI labeling kit (Miltenyi Biotech) according to the manufacturer’s instructions. Briefly, freshly isolated leukocytes were incubated with Annexin V-FITC for 15 min, washed for 10 min, then directly measured after addition of PI. Apoptosis rates of the neutrophil subset were shown through the gating strategy used (Supplementary Fig. 3). Annexin V binds to phospholipid phosphatidylserine, which is redistributed from intracellular to extracellular during apoptosis, whereas PI penetrates only cell membranes of dead or dying cells and binds to DNA.

The quantity of neutrophil ROS production was observed by DCF-DA (Merck, Darmstadt, Germany) staining. Blood cells (*n* = 10) and milk cells from healthy (*n* = 5) and inflamed (*n* = 7) udder quarters were incubated with 10 µM DCF-DA and measured before and after 90 min of stimulation with PMA (Sigma-Aldrich, Darmstadt, Germany; 1 µg/ml). ROS production of the neutrophil subset was shown through the applied gating strategy (Supplementary Fig. 4).

Measurements were performed with NovoCyte Quanteon flow cytometer (Agilent Technologies, Waldbronn, Germany). Data were analyzed using Flowlogic Software V7 (Inivai Technologies, Mentone Victoria, Australia) and NovoExpress Software version 1.5.0 (Agilent Technologies). Antigen expression intensity was calculated using mean fluorescence intensity values.

### Measurement of oxygen consumption rate and cell metabolic analysis by Seahorse XFe Analyzer

Quantification of leukocyte activation and metabolic phenotypes of blood cells (*n* = 4) and milk cells from healthy (*n* = 4) and inflamed (*n* = 4) udder quarters were determined by measuring the OCR using a Seahorse XFe Analyzer (Agilent Technologies). The Seahorse XFe Analyzer measures extracellular concentration changes of dissolved oxygen and free protons caused by cellular oxygen consumption and proton excretion. Following the manufacturer’s instructions, the isolated leukocytes from the blood and milk samples were seeded in 24-well XF24 cell culture microplates (Agilent Technologies) coated with 52 µl Poly-D-Lysin (Merck). Cells were seeded at a concentration of 1.5×10^6^ cells per well in sterile XF assay buffer (Seahorse XF RPMI medium supplemented with 10 mM glucose, 2 mM L-glutamine, and 1 mM pyruvate, pH 7.4; Agilent Technologies), while at least four wells were kept free from cells for background correction. The plate was briefly centrifuged at 500×g for one minute at room temperature and brake on to ensure that the cells settled evenly. Afterwards, cells were allowed to rest for 45 min at 37 °C. Prior to measurement, the four ports of the sensor cartridge plate (Agilent Technologies) were filled with the required compounds to be consecutively injected into the wells at defined timepoints. Prior to injection, baseline measurement was performed.

For quantification of neutrophil activation, mitochondrial inhibition was induced by injection of Rot/AA from port A (final well concentration: 0.5 µM). Subsequently, cells were stimulated by injection of PMA from port B (final well concentration: 100 ng/ml). OCR was measured every 6 min for 3 cycles for baseline measurement, for 5 cycles after Rot/AA injection and for at least 60 cycles after injection of PMA.

For determining the metabolic phenotypes via mitochondrial metabolism of leukocytes from blood and milk, the Agilent Seahorse XF Cell Mito Stress Test was used according to the manufacturer´s protocol (Agilent Technologies). This test targets the electron transport chain (ETC). Briefly, oligomycin was injected into the wells from port A for inhibition of ATP Synthase (complex V). FCCP was injected from port B for uncoupling of electron flow at the inner mitochondrial membrane. Rot/AA was injected from port C for inhibition of mitochondrial respiration via complex I and III of the ETC. Measurements were performed every 8.5 min for 5 cycles before / after compound injection.

Analyses were performed with at least two technical replicates for each cell fraction per individuum. Mean values of technical replicates were used for further statistical analysis. OCR was reported in units of pmol/min. Data analysis was done using WAVE 2.6 Software according to the manufacturer’s manual (Agilent Technologies).

### Statistical analysis

Data from all sampled cows were grouped into blood, healthy udder quarter and inflamed udder quarter. To compare differences between groups, the Kolmogorov-Smirnov (KS) test was used for the determination of Gaussian distribution. If the KS test indicated *p* ≤ 0.05 (no normal distribution), the Mann-Whitney test was used for statistical analysis. In case of normal distribution (KS test with *p* > 0.05), statistics were performed using student´s t-test. In both tests, the results were considered significant at *p* ≤ 0.05. Data were processed, analyzed and visualized with GraphPad Prism software (version 5.04, GraphPad Software, San Diego, CA, USA).

## Electronic supplementary material

Below is the link to the electronic supplementary material.

Supplementary Material 1

## Data Availability

The authors declare that the data supporting the findings of this study are available within the paper and its Supplementary files. Should any raw data be needed these are available from the corresponding author upon reasonable request.
