# Supplementary material for: Metabolic phenotype of bovine blood-derived neutrophils is altered in milk
Source: Sci Rep. 2025 Mar 19;15:9401. doi: 10.1038/s41598-025-93929-y (PMC11923088; doi:10.1038/s41598-025-93929-y)
Supplement: Supplementary file 1 — Supplementary Material 1 [file 41598_2025_93929_MOESM1_ESM.pdf]

Supplementary material

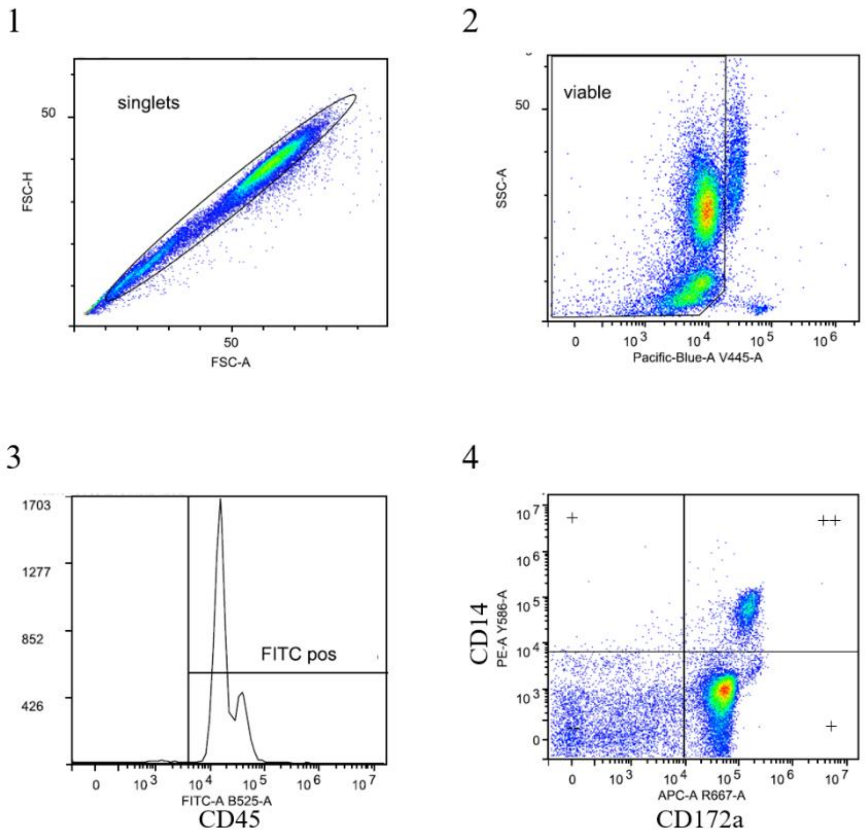

Supplementary Figure 1. Gating strategy for flow cytometry analysis on the example of $CD45^{+}CD172^{+}CD14^{+}$ cells. (1) Single cells were selected in a forward scatter-area vs forward scatter-height density plot. (2) Single cells showing viability dye were excluded. (3) Leukocytes were selected by $CD45^{+}$ expression. (4) $CD172a^{+}CD14^{+}$ cells were identified by CD172a vs CD14 density plot.

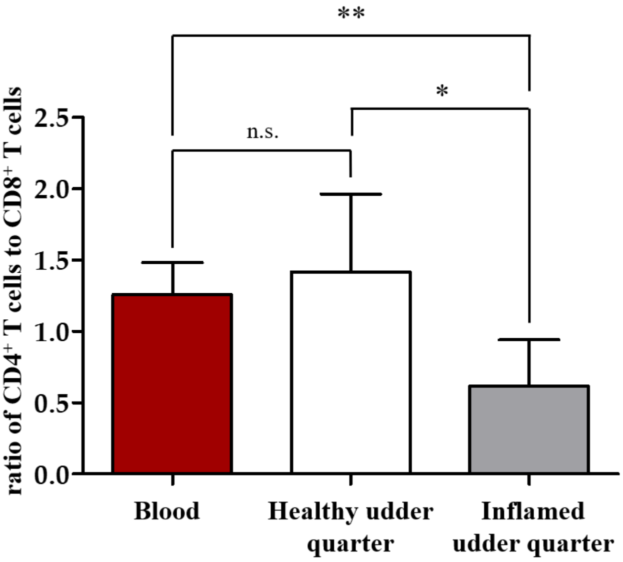

Supplementary Figure 2. Ratio of $CD4^+$ T cells to $CD8^+$ T cells. Milk-derived leukocytes from inflamed udder quarters showed a significantly (** $p \leq 0.01$, * $p \leq 0.05$) lower $CD4^+$:$CD8^+$ ratio compared to blood leukocytes and milk-derived leukocytes from healthy udder quarters.

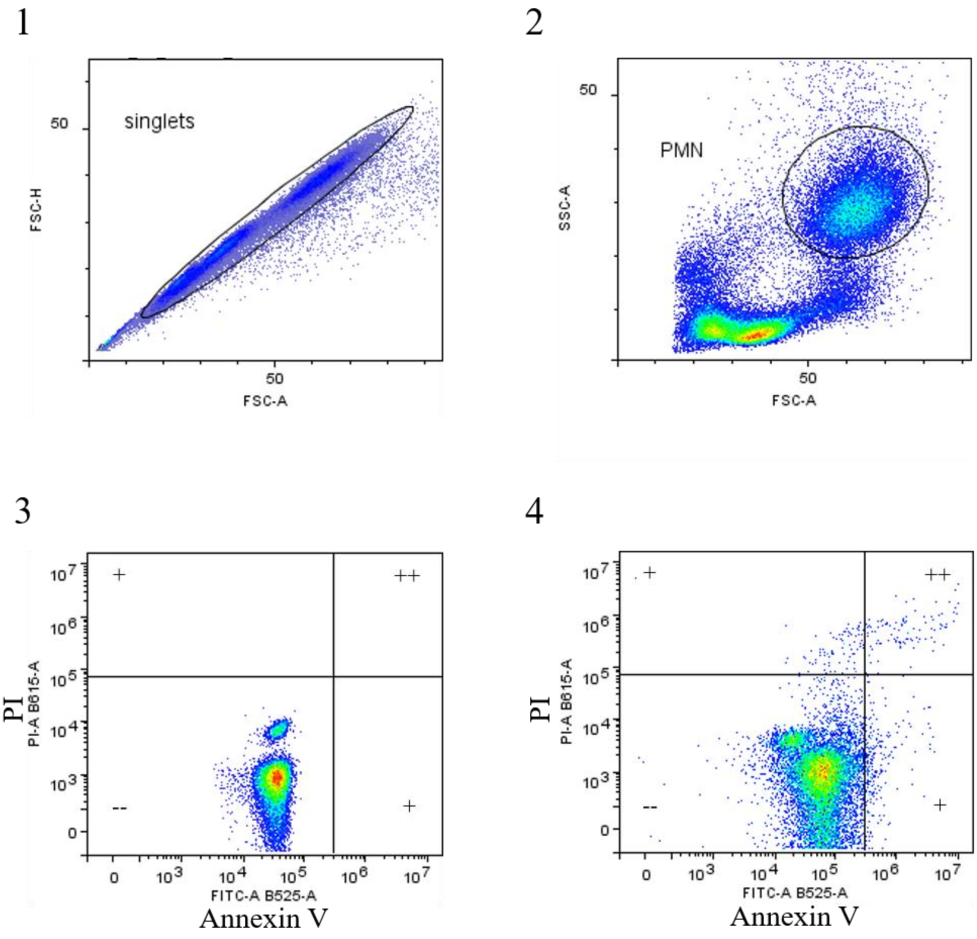

Supplementary Figure 3: Representative gating strategy for determination of apoptosis rate using flow cytometry analysis. (1) Single cells were selected in a forward scatter-area vs forward scatter-height density plot. (2) Neutrophils (PMN) were gated in a forward scatter-area vs side scatter-area density plot. (3) Unstained cells in Annexin V vs PI density plot showing no signal in the defined gate. (4) Annexin V/PI stained cells in Annexin V vs PI density plot.

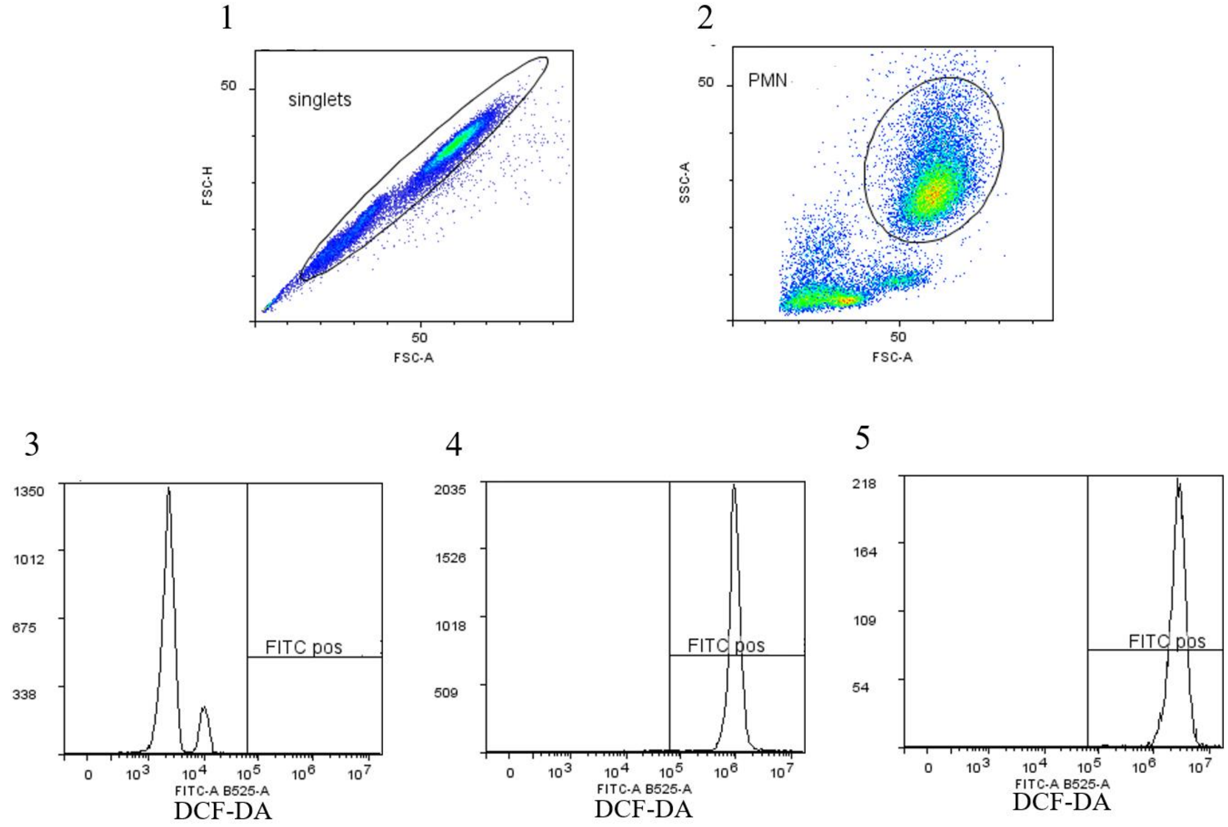

Supplementary Figure 4: Representative gating strategy for determination of ROS production using flow cytometry analysis. (1) Single cells were selected in a forward scatter-area vs forward scatter-height density plot. (2) Neutrophils (PMN) were gated in a forward scatter-area vs side scatter-area density plot. (3) Unstained cells showing no signal in the defined gate. (4) Unstimulated DCF-DA-stained cells. (5) DCF-DA-stained cells 90 min after PMA stimulation.
